# The aluminium-[^18^F]fluoride revolution: simple radiochemistry with a big impact for radiolabelled biomolecules

**DOI:** 10.1186/s41181-021-00141-0

**Published:** 2021-08-26

**Authors:** Stephen J. Archibald, Louis Allott

**Affiliations:** 1 Positron Emission Tomography Research Centre, Faculty of Health Sciences, University of Hull, Cottingham Road, Kingston upon Hull, HU6 7RX UK; 2 Department of Biomedical Sciences, Faculty of Health Sciences, University of Hull, Cottingham Road, Kingston upon Hull, HU6 7RX UK; 3 Hull University Teaching Hospitals NHS Trust, Castle Hill Hospital, Castle Road, Cottingham, HU16 5JQ UK

**Keywords:** Aluminium-[^18^F]fluoride, [^18^F]AlF, Radiometal, Chelation, Radioconjugate

## Abstract

The aluminium-[^18^F]fluoride ([^18^F]AlF) radiolabelling method combines the favourable decay characteristics of fluorine-18 with the convenience and familiarity of metal-based radiochemistry and has been used to parallel gallium-68 radiopharmaceutical developments. As such, the [^18^F]AlF method is popular and widely implemented in the development of radiopharmaceuticals for the clinic. In this review, we capture the current status of [^18^F]AlF-based technology and reflect upon its impact on nuclear medicine, as well as offering our perspective on what the future holds for this unique radiolabelling method.

## Introduction

**Fig. 1:**
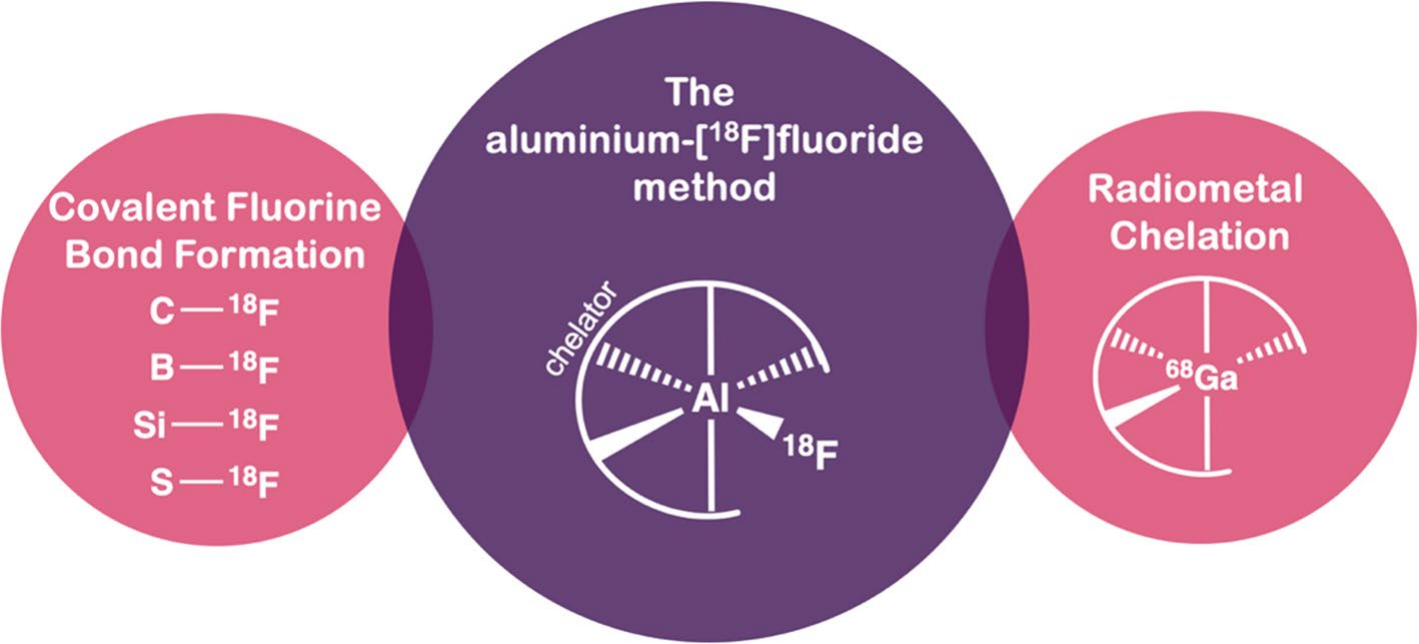
A schematic of general PET radiolabelling methods

The aluminium-[^18^F]fluoride ([^18^F]AlF) complex is a “pseudo-radiometal” which combines the favourable decay characteristics and scale of cyclotron produced fluorine-18 (t_1/2_ = 110 min, β_em_^+^ 0.635 MeV, 97%) with the convenience of metal-based radiochemistry (Fig. 1). The radiolabelling technique first described by McBride et al. (2009) went on to spark the imagination of the nuclear medicine community, and a decade later a diverse array of [^18^F]AlF-based radioconjugates, novel chelators, updated production methods including automated radiosynthesis, GMP compatible and compliant protocols and clinical trials have been reported. This review covers the latest advancements in the [^18^F]AlF radiolabelling method, including updates on the current status of the technique, [^18^F]AlF-radiopharmaceuticals in pre-clinical and clinical development, as well as areas for future development.

**Fig. 2:**
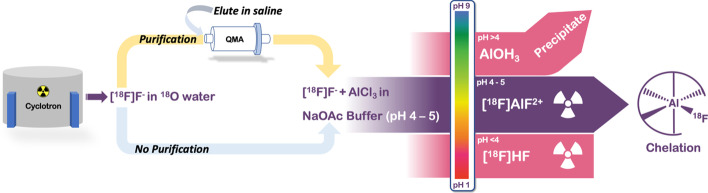
Schematic representation of the process for efficient [^18^F]AlF^2+^ complex formation and subsequent chelation from nucleophilic [^18^F]F^−^ in ^18^O water

The bond strength of aluminium-fluoride bond is ca. 670 kJ/mol and [^18^F]AlF forms thermodynamically stable and kinetically inert chelates (Smith et al. 2011; Farkas et al. 2015). The complex is formed by reacting AlCl_3_ with nucleophilic cyclotron produced (^18^O(*p,n*)^18^F) [^18^F]F^−^ in an aqueous milieu at pH ~ 4. Crucially, the formation of [^18^F]AlF is pH dependant with an optimal range between pH 4–5 (Fig. 2); more acidic conditions (pH < 4) favour the formation of [^18^F]HF and more basic (pH > 4) forming insoluble aluminium hydroxide species (Bruce Martin 1988; McBride et al. 2013).

The addition of an organic co-solvent increases the radiochemical yield (RCY) and is thought to better solubilise the [^18^F]AlF complex (D’Souza et al. 2011). The [^18^F]F^−^ anion can be used without purification in ^18^O water, but many opt to purify via quaternary methyl ammonium (QMA) solid-phase extraction (SPE) cartridges to remove unwanted metal ion impurities that may have been deposited from the cyclotron target which can potentially compete with [^18^F]AlF for coordination with the chelator (Table 1), as previously investigated for ^68^Ga radiochemistry (Šimeček et al. 2013). The trapping and release of [^18^F]F^−^ from a QMA cartridge is efficient (~ 99%), but interestingly Kersemans et al. (2018) observed that the choice of eluent (NaHCO_3_, NaNO_3_, NaCl or NaOAc) impacts the percentage of trapped activity eluted per volume unit which is an important consideration if a high radioactive concentration of [^18^F]F^−^ is required (Table 1, Entry **4**). The use of QMA SPE also concentrates [^18^F]F^−^ from large target volumes (mL vs. µL) to improve radiolabelling efficiency (He et al. 2014). As metal impurities can perturb efficient chelation, it is recommended that the highest purity reagents are used (e.g. AlCl_3_ 99.999% trace metals basis).

**Table 1:** Examples of [^18^F]F^−^ purification by QMA SPE cartridge

	Cartridge	Wash	Eluent	References
1	Sep-Pak light QMA	–	0.9% NaCl (1 mL)	Jiang et al. (2021)
2	Sep-Pak Accell Plus QMA	Water (5 mL)	0.05 M NaOAc, pH 4.5 (300 µL)	Lütje et al. (2019)
3	Sep-Pak light QMA	Water	0.5 M NaOAc, pH 4.5 (500 µL)	Giglio et al. (2018)
4	Sep-Pak Accell Plus QMA	Purged to dryness	0.5 M NaHCO_3_ *or* NaNO_3_ *or* NaCl *or* NaOAc (600 µL)	Kersemans et al. (2018)
5	Sep-Pak Accell Plus QMA light	Water (6 mL)	0.9% NaCl (250 µL)	Tshibangu et al. (2020)

Elution of [^18^F]F^−^ from a QMA is invariably efficient (ca. 99% recovery) but the choice of eluent can modulate radioactive concentration. This was demonstrated by Kersemans et al. (2018) and may be considered as a parameter for optimisation when developing new [^18^F]AlF labelling protocols (Kersemans et al. 2018)

**Fig. 3:**
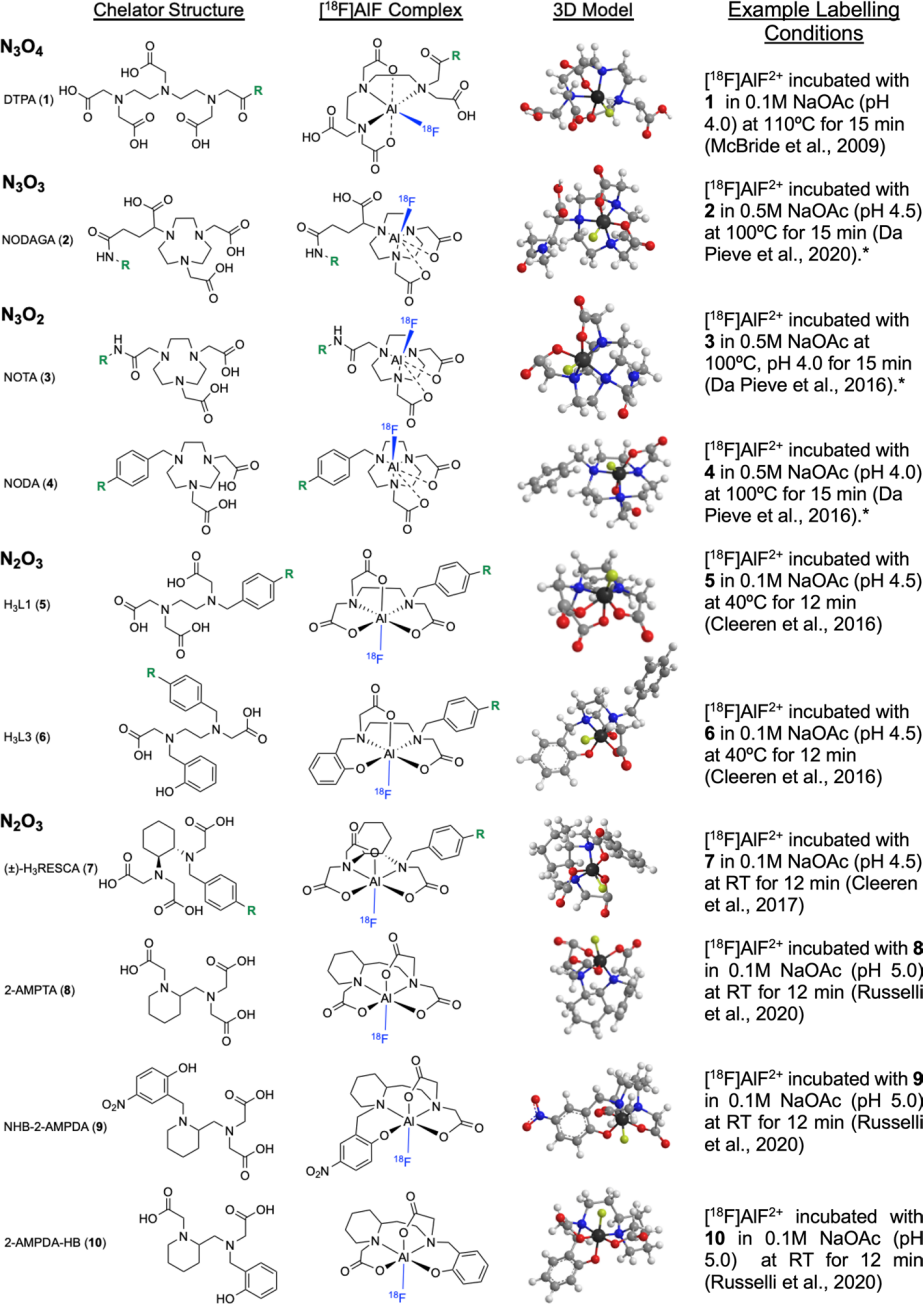
Structures of chelators evaluated for [^18^F]AlF and their proposed complexes. *R* = *bioconjugation handle*. 3D models were created in ChemBio3D (Cambridgesoft, UK) with MM2 energy minimization applied. Atom colours: carbon = light grey, hydrogen = white, oxygen = red, nitrogen = blue, fluorine = yellow, aluminium = dark grey. RT = room temperature. *Optional 1:1 (v/v) co-solvent included in the reaction mixture to improve RCY

Aluminium forms octahedral complexes but [^18^F]AlF^2+^ favours pentadentate ligands as one coordination site is already occupied by [^18^F]fluoride. The stability of an [^18^F]AlF-chelator complex in a radiopharmaceutical formulation (e.g. 0.9% saline) and under physiological conditions (37 °C in serum) is a determining factor of effective radiolabelling and subsequent radiopharmaceutical development. Diethylenetriaminepentaacetic acid (DTPA, **1**) with an N_3_O_4_ configuration was first evaluated as a chelator for [^18^F]AlF but the resulting complex exhibited poor in vitro stability in formulation and serum (McBride et al. 2009); however, the azamacrocyclic chelator 2,2′,2″-(1,4,7-triazacyclononane-1,4,7-triyl)triacetic acid (Fig. 3, NOTA, **3**) binding with an N_3_O_2_ donor set, formed stable chelates, evaluated in serum (4 h, 37 °C) (McBride et al. 2009). While NOTA chelates were very stable, the free carboxylate pendant arm competes with [^18^F]F^−^ for the aluminium coordination site and lowers the RCY (D’Souza et al. 2011; Shetty et al. 2011). NOTA and 1,4,7-triazacyclononane-1,4-diacetate (NODA, **4**) cyclic chelators binding with an N_3_O_2_ donor set form the most stable [^18^F]AlF complexes which form efficiently at elevated temperatures (100–120 °C); these are by far the most frequently used chelators in [^18^F]AlF radiopharmaceutical development owing to their commercial availability as both bifunctional chelators for conjugation to novel peptides, and as off-the-shelf chelator-peptide conjugates originally intended for use with ^68^Ga. There are numerous examples where the N_3_O_3_ configured NODAGA chelator (Fig. 3, **2**) has been radiolabelled with [^18^F]AlF, despite its unfavourable configuration leading to low RCY, to allow for comparative studies with a range of isotopes including ^64^Cu, ^68^Ga, ^111^In (Rylova et al. 2018; Eisenwiener et al. 2002).

As the [^18^F]AlF method has been applied to peptides previously radiolabelled with ^68^Ga, the relatively high temperatures (100–120 °C) required to chelate [^18^F]AlF^2+^ in N_3_O_2_ configured chelators is not problematic as small peptides have been proven withstand these temperatures. However, large proteins where biological activity is derived from a precise tertiary structure can denature at these temperatures, which has encouraged the development of [^18^F]AlF chelators for ambient temperature radiolabelling. Cleeren et al. (2016) described two promising acyclic chelators (Fig. 3, **5** and **6**) for [^18^F]AlF complexation at moderate temperatures (40 ºC) in good RCY (> 90%). While [^18^F]AlF-**6** showed stability in rat plasma at 2 h (ca 90%), a reasonable timeframe for PET imaging, it was poorly stable at 4 h (66%); [^18^F] AlF-**5** was unstable in vitro (Cleeren et al. 2016). This was improved by the N_2_O_3_ configured derivative (±)-H_3_RESCA (Fig. 3, **7**) which utilised a cyclohexyl moiety to impart structural rigidity into the molecule (Cleeren et al. 2017). (±)-H_3_RESCA was radiolabelled at room temperature and was stable in rat plasma for at least 4 h, comparable to NODA derivatives. [^18^F]AlF-(±)-H_3_RESCA conjugates of human serum albumin (RCY: 52–63%), nanobody NbV4m119 targeting CRIg (RCY: 35–53%) and an affibody molecule Z_HER2:2891_ targeting HER2 (RCY: 20 ± 7%) were synthesised, demonstrating the utility of this new chelator (Cleeren et al. 2017). Russelli et al. (2020) developed three acyclic chelators based around the 2-aminomethylpiperidine (AMP) group (Fig. 3, **8**–**10**). All chelators radiolabelled efficiently at room temperature and pH 5 (RCY: 55–81%) with 2-AMPTA-HB (**10**) showing the greatest stability at 240 min post radiosynthesis in human serum (87 ± 5%), PBS (93 ± 1%) and saline (92 ± 2%). The in vivo evaluation of [^18^F]AlF-2-AMPDA-HB showed low bone uptake at 2 h p.i. (1.63 ± 0.73%ID/g) (Russelli et al. 2020). The synthesis of a bifunctional derivative is now underway.

These new chelators provide an elegant solution to radiolabelling heat-sensitive biomolecules and will benefit from a full evaluation in the clinic; we predict that this will be achieved within the next five years. It is our opinion that the commercial availability and affordability of pentadentate NODA and NOTA derivatives mean they are unlikely to be replaced by acyclic chelators, at least for the time being, for instances where ambient temperature radiochemistry is a convenience rather than a necessity. If the research community adopt these chelators (or future derivatives) for use in projects, their commercialisation may be expedited. The development of ambient temperature kit-based production protocols, akin to ^68^Ga-trishydroxypyridinone (THP) which aims to be simple to use in the radiopharmacy setting (Young et al. 2017), may also increase the demand and implementation of alternative chelators. Nevertheless, it is important to remember that multistep synthesise of chelators can present a barrier to their use in projects which aim to focus on radioconjugate development.

## [^18^F]AlF-based radiopharmaceuticals

A range of [^18^F]AlF-based radiopharmaceuticals have been developed for variety of biological targets and some have transitioned into the clinic for evaluation in patients. A selection of prominent examples is discussed in this review and their production scale, radiochemical yield (RCY), molar activity (A_m_), status of automated radiosynthesis and clinical evaluation are summarised in Table 2; their structures, drawn in full where appropriate, are included in the relevant sections.

**Table 2:**
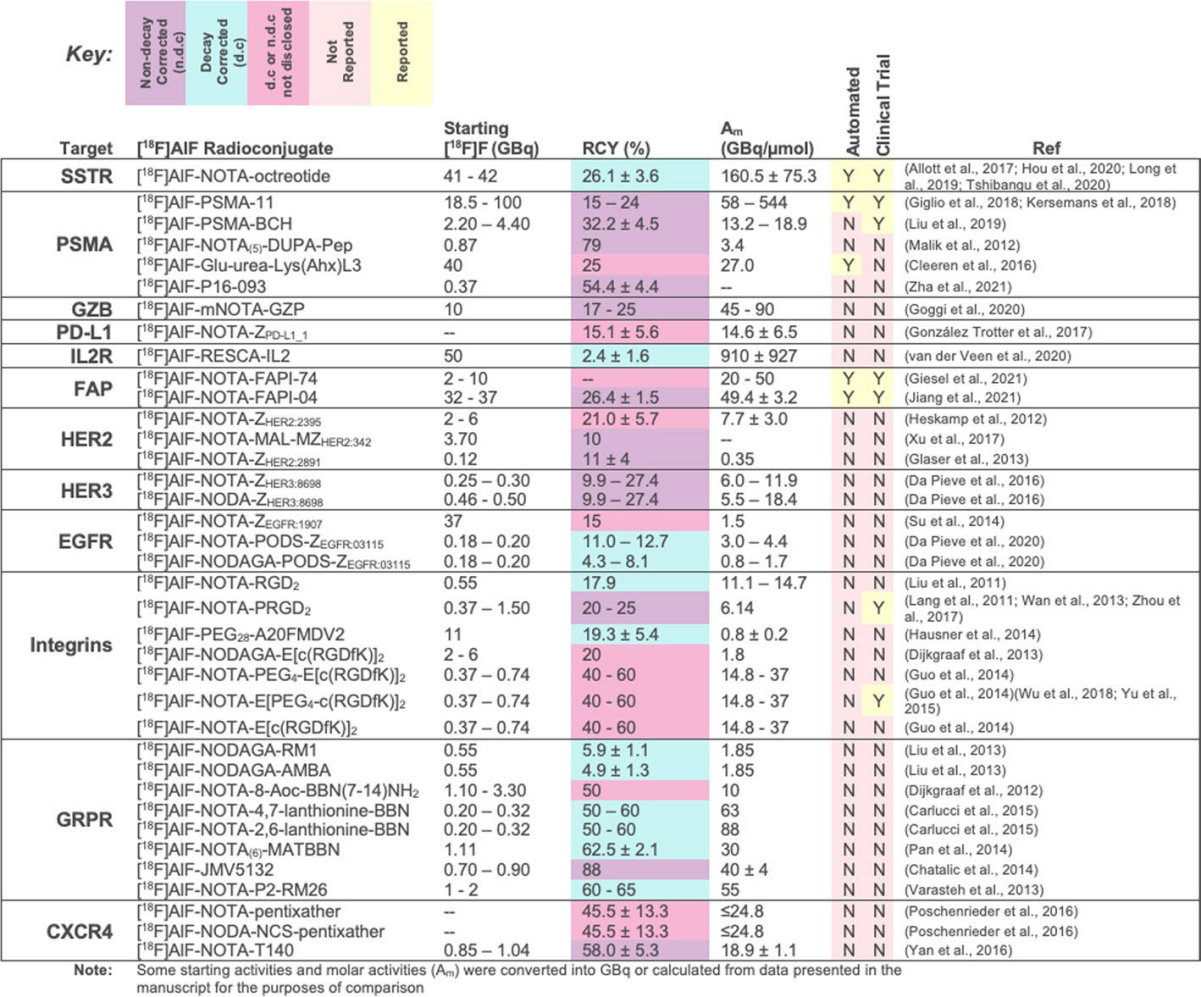
Prominent examples of [^18^F]AlF radioconjugates discussed in this review

### Imaging the somatostatin receptor (SSTR)

**Fig. 4:**
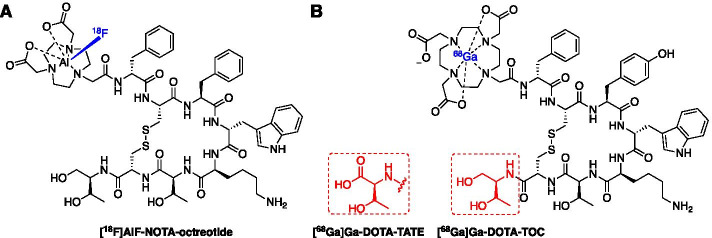
Structure of **A** [^18^F]AlF-NOTA-octreotide and **B** [^68^Ga]Ga-DOTA-TATE/[^68^Ga]Ga-DOTA-TOC

The NETTER-1 clinical trial successfully demonstrated that patients with neuroendocrine tumours (NETs) expressing somatostatin receptor type 2 (SSTR2) can be selected by PET scanning with radiopharmaceuticals [^68^Ga]Ga-DOTA-TATE (in the US) or [^68^Ga]Ga-DOTA-TOC (in Europe) (Fig. 4B) to receive [^177^Lu]Lu-DOTA-TATE (Lutathera™) peptide receptor radionuclide therapy (PRRT) (Strosberg et al. 2017); with outstanding clinical results for these patients, Lutathera™ has been approved by the Food and Drug Administration (FDA) and the European Medicines Agency (EMA) and realises the drive towards personalised medicine. With a likely rise in demand for somatostatin-based PET imaging over the next decade, many have looked towards fluorine-18 derivatives to increase capacity (Dubash et al. 2016; Allott et al. 2019, 2020; Maschauer et al. 2016; Ilhan et al. 2020; Waldmann et al. 2019). The [^18^F]AlF method was implemented to radiolabel a NOTA-conjugated octreotide in 2010 and preclinical evaluation of [^18^F]AlF-NOTA-octreotide showed high binding in vitro towards SSTR2 (Fig. 4A) (Laverman et al. 2010, 2012). Allott et al. (2017) first described the automated radiosynthesis of [^18^F]AlF-NOTA-octreotide using the GE TRACERLab™ FX_FN_ and Trasis AllInOne (AIO)™ platforms and Tshibangu et al. (2020) advanced this work by reporting a fully GMP compliant production on the Trasis AllInOne™ platform; [^18^F]AlF-NOTA-octreotide was produced in a 26.1 ± 3.6% radiochemical yield (d.c.) with an apparent molar activity of 160.5 ± 75.3 GBq/µmol within 40 min (Tshibangu et al. 2020).

The clinical evaluation of [^18^F]AlF-NOTA-octreotide in three healthy volunteers and 22 patients with neuroendocrine neoplasms (NEN) was well tolerated and in patients with NEN, high tumour uptake and tumour-to-background ratios were observed (Long et al. 2019). Pauwels et al. (2019) reported a clinical comparison of [^68^Ga]Ga-DOTA-TATE and [^18^F]AlF-NOTA-octreotide in a patient with diffuse metastases of a rectal NET which concluded that both radiopharmaceuticals produced comparable results with improved contrast in multiple smaller lesions. Further head-to-head comparison of [^18^F]AlF-NOTA-octreotide and [^68^Ga]Ga-DOTA-TATE in patients with NEN showed no significant difference between uptake in most organs showed and a similar image quality; however, uptake of [^18^F]AlF-NOTA-octreotide was lower in the liver which benefited the detection of lesions in this organ (Hou et al. 2020). All studies taken together, [^18^F]AlF-NOTA-octreotide appears to be a promising alternative to [^68^Ga]Ga-DOTA-TATE and has the potential for centralised, to increase availability and lower costs. All clinical trials of [^18^F]AlF-NOTA-octreotide are presented in Table 3.

**Table 3:** Clinical PET studies using [^18^F]AlF-NOTA-octreotide

Year	Study	N^o^ Participants	Outcome	References
[^18^F]AlF-NOTA-octreotide				
2019	First comparison with [^68^Ga]Ga-DOTATATE in diffuse metastases of rectal NET	1	[^18^F]AlF-NOTA-octreotide was a promising clinical alternative for [^68^Ga]Ga-DOTATATE and warrants further head-to-head evaluation	Pauwels et al. (2019)
2019	Biodistribution, safety and dosimetry in healthy volunteers. Detection of neuroendocrine neoplasms (NENs) in patients	Healthy: 3 Patients: 22	[^18^F]AlF-NOTA-octreotide was well tolerated and provided superior imaging of well-differentiated NENs. Tumour-to-background ratios were significantly higher compared to [^18^F]FDG	Long et al. (2019)
2020	Biodistribution, dosimetry and comparison with [^68^Ga]Ga-DOTATATE in NETs	Healthy: 6 Patients: 6	[^18^F]AlF-NOTA-octreotide was safe and well tolerated. Lesion detection rate and tumour-to-background ratios were comparable to [^68^Ga]Ga-DOTATATE	Pauwels et al. (2020)
2020	Head-to-head comparison with [^68^Ga]Ga-DOTATATE in patients with NENs	Healthy: 3 Patients: 8	[^18^F]AlF-NOTA-octreotide produces similar image quality and fine detection rate of lesions, especially in the liver, because of lower liver background uptake than [^68^Ga]Ga-DOTATATE	Hou et al. (2020)

### Imaging prostate specific membrane antigen (PSMA)

Prostate specific membrane antigen (PSMA) is highly expressed in prostate cancer (PCa) and, when imaged by targeted PET radioligands, provides a crucial biomarker to assess disease burden with the potential for patient stratification to receive PRRT (Werner et al. 2020; Rahbar et al. 2017; Sathekge et al. 2019). Many radiopharmaceuticals have been developed around the simple Lys-Urea-Glu binding motif and linker strategies have been adapted to modulate binding affinity and PK. ^68^Ga-based PSMA radiopharmaceuticals have been extensively evaluated in the clinic, leading to FDA approval for [^68^Ga]Ga-PSMA-11 in December 2020 for PET imaging of PSMA positive lesions in men with prostate cancer. Fluorine-18 PSMA radioligands have been developed and evaluated clinically, including [^18^F]DCFPyL, [^18^F]PSMA-1007, [^18^F]JK-PSMA-7 and [^18^F]CTT1057; while their structures differ, they share a similar radiolabelling approach based around the ^18^F-fluorination of an aromatic moiety (Werner et al. 2020; Bouvet et al. 2016; Naka et al. 2020; Zlatopolskiy et al. 2019; Behr et al. 2019).

**Fig. 5:**
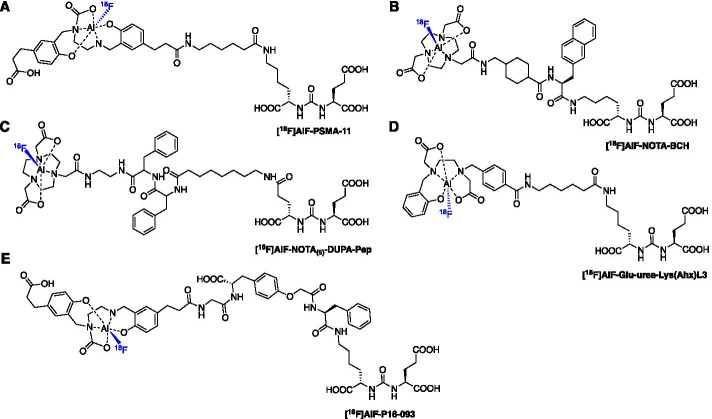
Structures of **A** [^18^F]AlF-PSMA-11, **B** [^18^F]AlF-PSMA-BCH, **C** [^18^F]AlF-NOTA_(5)_-DUPA-Pep, **D** [^18^F]AlF-Glu-urea-Lys(Ahx)L3 and **E** [^18^F]AlF-P16-093

[^18^F]AlF-based PSMA radioligands have been developed with [^18^F]AlF-PSMA-11, a derivative of [^68^Ga]Ga-PSMA-11, being the most clinically advanced. The synthesis of [^18^F]AlF-PSMA-11 was described by Malik et al. (2015) and Boschi et al. (2016), then first produced in a fully automated radiosynthesis using the GE TRACERlab™ FX_FN_ platform in a RCY of 18 ± 3% (n.d.c) and a RCP > 95 ± 3% (Fig. 5A) (Giglio et al. 2018; Boschi et al. 2016; Malik et al. 2015). An automated method was developed for the SyntheraFCHOL™ module which produced large scale batches (24.0 ± 6.0 GBq) of [^18^F]AlF-PSMA-11 in a RCY of 21 ± 3% and RCP > 95%; batch stability was confirmed for 4 h and conformed to European Pharmacopeia guidelines (Kersemans et al. 2018). PSMA-11 bears the N,N-bis(2-hydroxybenzyl)ethylenediamine-N,N-diacetic acid (HBED) chelator which is unfavourably configured (N_2_O_4_) for chelating the [^18^F]AlF complex, however the stability of the final formulated dose was confirmed up to 4 h and [^18^F]AlF-PSMA-11 was stable in human plasma up to 1 h (Giglio et al. 2018). Lütje et al. (2019) further evaluated the radioligand in PSMA-expressing xenografts alongside [^68^Ga]Ga-PSMA-11, but showed that formulation composition strongly influenced the stability of the [^18^F]AlF chelate. [^18^F]AlF-PSMA-11 was not stable in water (64.5% RCP after purification) and while stability was greater in NH_4_OAc (25 mM, pH 6.9), a decrease in RCP was observed from 98.5 to 92.5% over 180 min (Lütje et al. 2019). Regardless, [^18^F]AlF-PSMA-11 vs. [^68^Ga]Ga-PSMA-11 uptake in PSMA expressing LS174T-PSMA tumours was highest at 2 h p.i. (10.8 ± 2.3 vs 7.9 ± 1.3%ID/g), uptake in the bone was 5.0 ± 0.6 vs 0.1 ± 0.0%ID/g and renal uptake of [^18^F]AlF-PSMA-11 was lower than [^68^Ga]Ga-PSMA-11 (Lütje et al. 2019). A similar study by Piron et al. (2020a) reported no significant increase in bone uptake over 2 h p.i., with the highest uptake observed in the humerus and skull at 2 h p.i. (1.96 and 1.94%ID/g).

The radiation safety and dosimetry of [^18^F]AlF-PSMA-11 was evaluated in six patients with suspected PCa recurrence after previous treatment. The mean effective dose was 12.8 ± 0.6 µSv/MBq, which was lower than [^68^Ga]Ga-PSMA-11, and the radioligand highlighted suspected metastatic disease (Piron et al. 2019). An increase of 22.2 ± 1.5% of [^18^F]fluoride was measured in plasma 90 min p.i. but this did not translate into extensive bone uptake (Piron et al. 2019). The PET protocol was later optimized in a larger study of 44 patients (Piron et al. 2020b). An intraindividual comparison between [^18^F]AlF-PSMA-11 and [^68^Ga]Ga-PSMA-11 in prostate cancer patients with biochemical relapse concluded that both radioconjugates have similar and clinically relevant diagnostic value (Santos et al. 2020). The clinical studies have been summarised in Table 4.

**Table 4:** Clinical PET studies using

Radioconjugate and Year	Study	N^o^ Participants	Outcome	References
[^18^F]AlF-PSMA-BCH (2019)	Newly diagnosed prostate cancer (PCa)	11	Tumours identified	Liu et al. (2019)
[^18^F]AlF-PSMA-11 (2019)	Dosimetry and biodistribution in PCa	6	Radioconjugate can be safely administered with a mean effective dose of 12.8 ± 0.6 µSv/MBq, similar to [^18^F]DCFPyL	Piron et al. (2019)
[^18^F]AlF-PSMA-11 (2020)	Optimisation of PET protocols for PCa	44	2.0 ± 0.2 MBq/kg identified as the preferred dose. Diuretic can be useful for lesions in proximity to the ureters	Piron et al. (2020b)

Two NOTA derivatives containing the Lys-Urea-Glu PSMA binding motif have been developed ([^18^F]AlF-NOTA_(5)_-DUPA-Pep and [^18^F]AlF-PSMA-BCH, Figs. 5B, 5C) which differ in their linker strategy. The radioconjugates were synthesised in a RCY of 79 ± 0.7% and 32.2 ± 4.5%, respectively (Liu et al. 2019; Malik et al. 2012). While [^18^F]AlF-NOTA_(5)_-DUPA-Pep is yet to be preclinically evaluated, [^18^F]AlF-PSMA-BCH entered a small clinical trial of 11 newly diagnosed PCa patients; the radioconjugate was well tolerated and visualised tumour lesions at 1 and 2 h p.i. (Liu et al. 2019). Cleeren et al. (2016) developed [^18^F]AlF-Glu-urea-Lys(Ahx)L3 to exemplify the application of their new [^18^F]AlF chelator that reacts efficiently at lower temperatures (Fig. 5D). The radioconjugate was produced in a large batch (8.14 GBq) from a 12 min reaction at 40 ºC; the RCY was 25% (unoptimized) and the molar activity was 27 GBq/µmol (Cleeren et al. 2016). No significant bone uptake was observed at 60 min p.i. (0.74 ± 0.07% %ID/g), encouraging the further development of more efficient chelators that react at lower temperatures, ultimately leading to (±)-H_3_RESCA (Cleeren et al. 2016, 2017). Zha et al. (2021) described the synthesis and pre-clinical evaluation of [^18^F]AlF-P16-093, derived from [^68^Ga]Ga-P16-093, a PSMA targeted radioligand currently in phase II clinical trial. [^18^F]AlF-P16-093 showed high in vivo tumour uptake in mouse PIP-PC3 xenografts (18.8 ± 5.14%ID/g at 60 min p.i.) with some uptake in the bone (2.82 ± 0.49%ID/g), higher than observed for [^68^Ga]Ga-P16-093 (0.26 ± 0.07%ID/g), indicating demetallation (Zha et al. 2021).

### Imaging the immune system

Immune checkpoint inhibitors are an important therapeutic intervention for tumours evading the immune system, but patient response is variable (Postow et al. 2015; Royal et al. 2010; Marrone et al. 2016). Immune checkpoints PD-1 and CTLA-4 are often targeted in combination to improve response rates but can lead to toxicity and immune-related side effects (Khoja et al. 2017; Cousin and Italiano 2016). Radiopharmaceuticals to measure treatment response are of great interest and the [^18^F]AlF method has been used to develop radioconjugates for imaging T-cell activation.

**Fig. 6:**
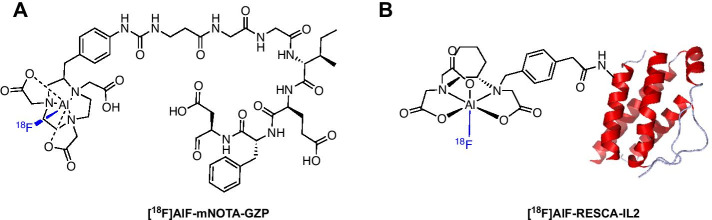
Structure of **A** [^18^F]AlF-mNOTA-GZP and **B** [^18^F]AlF-RESCA-IL2

Granzyme B is a serine protease released upon the activation of cytotoxic T cells and has been targeted by a gallium-68 labelled peptide [^68^Ga]Ga-NOTA-GZP (Fig. 6A) (Larimer et al. 2017). Preclinical imaging distinguished between responders and non-responders to monotherapy (anti-PD-1) and combination immunotherapy (anti-PD-1 and anti-CTLA-4) with excellent predictive ability (Larimer et al. 2017; Goggi et al. 2020b). Goggi et al. (2020a) radiolabelled the peptide with the [^18^F]AlF complex to improve PET sensitivity and spatial resolution. Formulated [^18^F]AlF-mNOTA-GZP was produced in 50 min from [^18^F]fluoride in a 17–25% RCY (n.d.c) and a molar activity of 45–90 GBq/μmol. The enzyme inhibition efficiency of both ^68^Ga and [^18^F]AlF peptides were similar. The uptake of [^18^F]AlF-mNOTA-GZP correlated with changes in T cell populations and distinguished responders and non-responders to monotherapy and combination immunotherapy (Goggi et al. 2020a). An automated radiosynthesis of [^18^F]AlF-mNOTA-GZP has not yet been described.

The interleukin-2 receptor (IL2R) is overexpressed on activated T cells and PET radioligands have been developed using recombinant IL2 as a targeting molecule. Gialleonardo et al. (2012) first labelled IL2 with fluorine-18 via the [^18^F]SFB prosthetic group ([^18^F]FB-IL2) and the radiosynthesis was translated into a GMP compliant clinical production to evaluate [^18^F]FB-IL2 in human trials (Veen et al. 2019). The complexity of automating the radiosynthesis of [^18^F]FB-IL2 encouraged the simplification of the radiolabelling process by employing the [^18^F]AlF method and the RESCA chelator to produce [^18^F]AlF-RESCA-IL2 (Fig. 6B) (Veen et al. 2020). The radioconjugate was synthesised in a 2.4 ± 1.6% RCY with a molar activity of 910 ± 927 GBq/µmol in multipatient doses (1375 ± 791 MBq from < 50 GBq fluorine-18) in 90 min (Veen et al. 2020). [^18^F]AlF-RESCA-IL2 showed high in vitro uptake in activated peripheral blood mononuclear cells (PBMC), and in vivo uptake in PBMC xenografts and lymphoid tissue, supporting further evaluation of this radioligand.

An [^18^F]AlF-based radioconjugate was developed to target programmed death ligand 1 (PD-L1), which is expressed in tumours responding to anti-PD-1 therapy (e.g. pembrolizumab). The affibody molecule PD-L1 (Z_PD-L1_1_) was conjugated to NOTA via its unique cystine and radiolabelled with the [^18^F]AlF complex ([^18^F]AlF-NOTA-Z_PD-L1_1_ in a radiochemical yield of 15.1 ± 5.6% and a molar activity of 14.6 ± 6.5 GBq/µmol; the radioconjugate differentiated between PD-L1 positive and negative tumours in vivo (González Trotter et al. 2017).

### Imaging fibroblast activation protein (FAP)

**Fig. 7:**
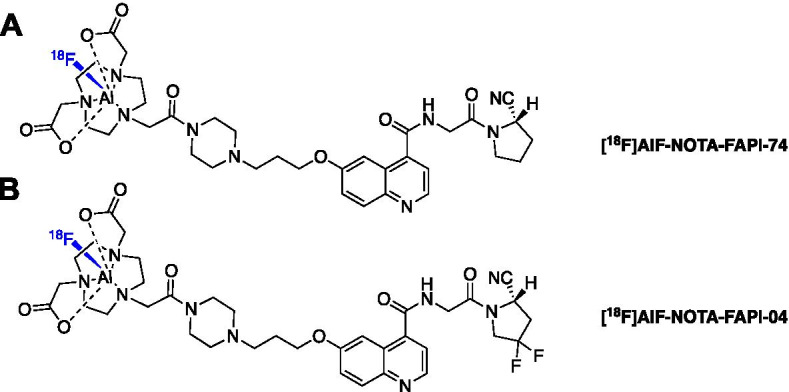
Structure of **A** [^18^F]AlF-NOTA-FAPI-74 and **B** [^18^F]AlF-NOTA-FAPI-04

Fibroblast activation protein (FAP) is highly expressed in many human cancers and can be targeted by quinoline-based FAP inhibitors (FAPIs) (Brennen et al. 2012; Jansen et al. 2013). Gallium-68 FAP radioligands have been evaluated and exhibit excellent tumour contrast (Kratochwil et al. 2019; Giesel et al. 2019). Given the promise for a radiolabelled FAPI entering routine clinical practice, the potential future demand could outstrip the supply of generator-produced gallium-68. The [^18^F]AlF method was employed to produce a fluorine-18 derivative with the view of centralised large-scale production (Giesel et al. 2021). FAPI-74 bears a NOTA chelator and efficiently radiolabelled with the [^18^F]AlF complex starting from 2 to 10 GBq of fluorine-18 (Fig. 7A). [^18^F]AlF-NOTA-FAPI-74 was evaluated in 10 patients with lung cancer and showed high contrast and low radiation burden (Giesel et al. 2021). This example highlights the synergy between ^68^Ga and [^18^F]AlF radioconjugates, and the simplicity in transforming from one isotope to another if an appropriate chelator has been employed in the conjugate. It is important to recognise the importance of chelator selection in developing gallium-68 radioconjugates, which is elegantly illustrated with FAPI-74, whereby thinking forward to potential future scalability beyond gallium-68 encouraged the use of the NOTA chelator; had DOTA or any other gallium-68 specific chelator being selected, then exploring the [^18^F]AlF method would have been non-trivial.

The centralised production of [^18^F]AlF-based radiopharmaceuticals requires automated radiosynthesis and Jiang et al. (2021) describe the production of [^18^F]AlF-NOTA-FAPI-04 on a cassette-based Trasis AIO™ platform (Fig. 7B). Multipatient doses of radiopharmaceutical were synthesised (9.1 ± 0.6 GBq) in an excellent RCY of 26.4 ± 1.5% (n.d.c) which enabled both preclinical in vivo evaluation and clinical scan (Jiang et al. 2021).

### Imaging the epidermal growth factor (EGF) receptor family

The epidermal growth factor (EGF) receptor family are overexpressed in many cancers and therapeutics have been developed towards these targets, including Cetuximab targeting EGFR and Trastuzumab targeting HER2 (Wang 2017; Xu et al. 2017b; Pernas and Tolaney 2019). Stratifying patients based on EGF receptor expression using PET imaging is of great interest and while zirconium-89 labelled monoclonal antibodies (mAb) with long-lived radioisotopes (t_1/2_ = 3.3 days) have been developed, smaller biomolecule fragments with fast PK and radiolabelled short half-life isotopes are of great interest (Tolmachev and Orlova 2020).

The first Affibody molecule to be radiolabelled with the [^18^F]AlF complex was Z_HER2:2395_ which targeted the HER2 receptor and showed specific tumour uptake (T:B of 7.4 ± 1.8 and tumour uptake of 4.4 ± 0.8%ID/g at 1 h p.i.) in SK-OV-3 xenografts (Heskamp et al. 2012). A NOTA-maleimide chelator was conjugated to the unique cystine residue of the Affibody molecule and radiolabelled within 30 min to produce [^18^F]AlF-NOTA-Z_HER2:2395_ in a RCY of 21.0 ± 5.7% and molar activity of 7.7 ± 3.0 GBq/µmol; the affinity of the radioconjugate for HER2 was K_d_ = 6.2 nM (Heskamp et al. 2012). A similar approach was taken to produce [^18^F]AlF-NOTA-MAL-MZ_HER2:342_ and [^18^F]AlF-NOTA-Z_HER2:2891_ (Xu et al. 2017a; Glaser et al. 2013).

**Fig. 8:**
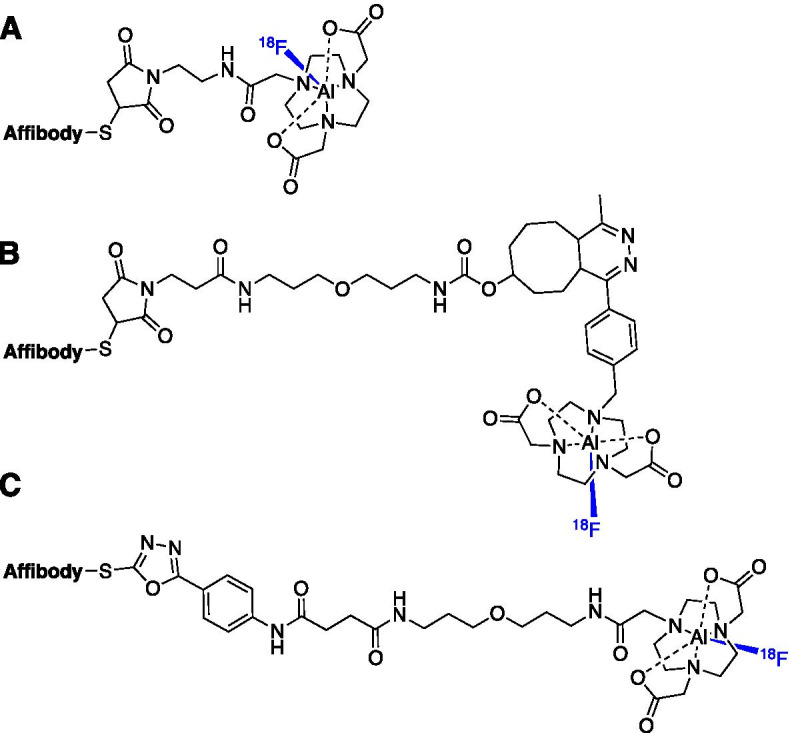
Three different approaches to radiolabelling Affibody molecules with the [^18^F]AlF complex: **A** maleimide-thiol conjugation; **B** IEDDA “click” approach and **C** PODS conjugation

A HER3 targeted affibody molecule Z_HER3:8698_ was radiolabelled with the [^18^F]AlF complex using two methods: (1) direct [^18^F]AlF chelation with NOTA-Z_HER3:8698_ and (2) a prosthetic group approach using inverse electron demand Diels–Alder (IEDDA) chemistry (Pieve et al. 2016). Approach 1 produced [^18^F]AlF-NOTA-Z_HER3:8698_ in a RCY of 9.9–27.4% (n.d.c) and molar activity of 6.0–11.9 GBq/µmol (Fig. 8A). In approach 2, a NODA conjugated tetrazine (NODA-Tz) was synthesised alongside a TCO-conjugated Affibody molecule (Fig 8B). [^18^F]AlF-NODA-Tz was synthesised independently of the Affibody molecule and subsequently incubated with TCO-Z_HER3:8698_ at an ambient temperature, producing a conjugate with a higher molar activity but over a larger range (5.5–18.4 GBq/µmol) (Pieve et al. 2016). This strategy exemplifies a method for radiolabelling biomolecules with the [^18^F]AlF-NODA complex that may be sensitive to the high temperatures required for direct [^18^F]AlF chelation.

An EGFR targeted Affibody molecule [^18^F]AlF-NOTA-Z_EGFR:1907_ has been developed and produced in a radiochemical yield of 15% (Su et al. 2014). As all of these examples show, Affibody molecules are typically functionalised at their unique cystine residue via a maleimide-thiol Michael addition; however, instability has been observed during radiosynthesis and in vivo studies (Pieve et al. 2020; Ponte et al. 2016). Da Pieve et al. (2020) have recently described phenyloxadiazolyl methylsulfone (PODS) derivatives of NOTA and NODAGA chelators which provides an alternative to maleimide chemistry and may overcome instability associated with the maleimide-thiol ligation at later time points. [^18^F]AlF-NOTA-PODS-Z_EGFR:03115_ and [^18^F]AlF-NODAGA-PODS-Z_EGFR:03115_ radioconjugates were synthesised and showed specific tumour uptake 14.1 ± 5.3 and 16.7 ± 4.5%ID/g (1 h p.i.), respectively (Pieve et al. 2020).

Despite implementing the [^18^F]AlF method in the development of numerous Affibody molecule radioconjugates targeting the EGF family, the clinical translation of these probes is yet to be reported. However, given that ^68^Ga-labelled Affibody molecules targeting HER2 have been evaluated in phase I/II clinical trials, we are likely to see [^18^F]AlF derivatives in the near future (Sandström et al. 2016; Sörensen et al. 2016).

### Imaging integrins

**Fig. 9:**
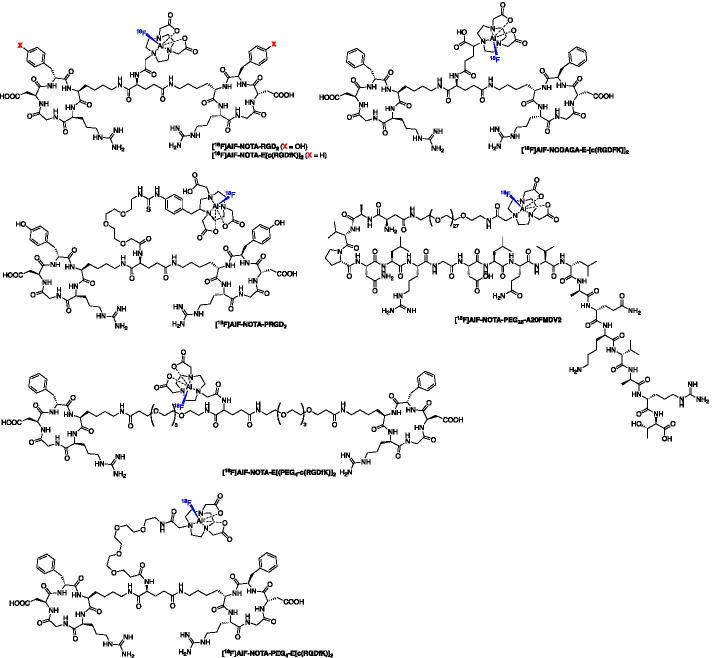
Structure of [^18^F]AlF labelled peptides targeting integrins

The integrins are a family of transmembrane receptors of which ⍺_v_β_3_ is involved in tumorigenesis and metastasis, making it an excellent target for PET imaging (Hamidi and Ivaska 2018). An arginine-glycine-aspartic acid (RGD) peptide sequence binds to integrins and forms the basis of many PET probes for imaging ⍺_v_β_3_. One of the first [^18^F]AlF-based RGD radioligand was reported by Liu et al. (2011) using a NOTA conjugated dimeric cyclic RGD peptide E[c(RGDyK)]_2_ (NOTA-RGD_2_). The [^18^F]AlF-NOTA-RGD_2_ peptide was produced in a RCY of 17.9% (d.c.) and molar activity of 11.1–14.8 GBq/µmol and showed high tumour uptake (5.3 ± 1.7%ID/g) and good T:M contrast at 60 min p.i. (Fig. 9) (Liu et al. 2011). Lang et al. (2011) developed [^18^F]AlF-NOTA-PRGD_2_ (also known as ^18^F-Alfatide), an [^18^F]AlF labelled derivative of [^18^F]FPPRGD which allowed for a more convenient radiosynthesis with comparable or superior imaging and PK properties. [^18^F]AlF-NOTA-PRGD_2_ was successfully evaluated in lung cancer patients and produced from a lyophilised kit within 20 min with a RCY (d.c.) of 42.1 ± 2.0% (Wan et al. 2013). A range of clinical studies evaluating [^18^F]AlF-NOTA-PRGD_2_ have been summarised in Table 5.

**Table 5:** Clinical PET studies using [^18^F]AlF-NOTA-PRGD_2_ (^18^F-Alfatide) and [^18^F]AlF-NOTA-E[PEG4-c(RGDfK)]_2_ (^18^F-Alfatide II)

Year	Study	N^o^ Participants	Outcome	References
[^18^F]AlF-NOTA-PRGD_2_ (^18^F-Alfatide)				
2013	Lung cancer	9	All tumours visualised	Wan et al. (2013)
2014	Differentiated thyroid cancer (DTC)	20	Most lymph node metastases showed abnormal uptake of the radioconjugate, but the diagnostic value was inferior to [^18^F]FDG	Cheng et al. (2014)
2015	Lung cancer	26	Successfully differentiated malignant lesions from hamartoma. Challenging to differentiate inflammatory or inflammatory pseudotumours from malignancy	Gao et al. (2015)
2016	Advanced non-small cell lung cancer (NSCLC)	18	May predict short-term outcome of concurrent chemoradiotherapy in patients with advanced NSCLC	Luan et al. (2016)
2016	Glioblastoma (GBM)	25	Visualisation of GBM and predictive of sensitivity to concurrent chemoradiotherapy as early as 3 weeks after treatment	Zhang et al. (2016)
2017	Lymph node metastases in NSCLC	13	Highly sensitive, specific and accurate detection of metastatic lymph nodes for NSCLC patients	Zhou et al. (2017)
2019	Esophageal Squamous Cell Carcinoma (ESCC)	61	No significant differences in uptake between ^18^F-Alfatide and [^18^F]FDG were observed, but may provide a complementary information about ESCC metastasis	Dong et al. (2019)
2019	Response to apatinib	38	High uptake in tumours correlated with a better response to apatinib therapy and may be of predictive value	Li et al. (2019)
[^18^F]AlF-NOTA-E[PEG_4_-c(RGDfK)]_2_ (^18^F-Alfatide II)				
2015	Healthy Volunteers	5	Well tolerated with no serious adverse events	Yu et al. (2015)
2015	Brain metastases	9	All brain lesions were visualised	Yu et al. (2015)
2015	Bone metastasis	30	Sensitivity in osteoblastic metastases was low but significantly higher than for [^18^F]FDG	Mi et al. (2015)
2018	Breast Cancer	44	Good performance but not superior to [^18^F]FDG in identifying breast cancer in this study. May have superiority in detecting strongly ER + ve and HER2-ve expression	Wu et al. (2018)
2018	Lung cancer & Tuberculosis (TB)	20	The radioconjugate was able to differentiate between lung cancer and TB	Du et al. (2018)

Guo et al. (2014) compared two novel dimeric RGD peptides, [^18^F]AlF-NOTA-PEG_4_-E[c(RGDfK)]_2_ and [^18^F]AlF-NOTA-E[PEG_4_-c(RGDfK)]_2_ which included PEG_4_ linker strategies aimed to optimise radiolabelling and PK performance of the probes and compared these to [^18^F]AlF-NOTA-RGD_2_. All probes were synthesised in a RCY of 40–60%, with molar activities ranging from 14.8 to 37 GBq/µmol. All three probes exhibited favourable in vivo performance with high tumour uptake and good target-to-background ratios, but [^18^F]AlF-NOTA-E[PEG_4_-c(RGDfK)]_2_ (also known as ^18^F-Alfatide II) was highlighted as a promising candidate for clinical translation owed to the lowest liver uptake and highest tumour uptake (2.92 ± 0.40%ID/g) (Guo et al. 2014). First-in-human studies of ^18^F-Alfatide II commenced in 2015 in healthy volunteers and patients with brain metastases; the radioconjugate was well tolerated in all healthy volunteers and successfully visualised all 20 brain lesions (Yu et al. 2015). A preliminary clinical study using ^18^F-Alfatide II to identify breast cancer was performed in 2018, concluding its suitability for clinical use in breast cancer patients and its good performance, albeit not superior to [^18^F]FDG in this study (Wu et al. 2018); a range of clinical studies evaluating [^18^F]AlF-NOTA-E[PEG_4_-c(RGDfK)]_2_ have been summarised in Table 5. Dijkgraaf et al. (2013) evaluated NODAGA-E[c(RGDfK)]_2_ for radiolabelling with multiple isotopes including [^18^F]AlF, ^68^Ga and ^111^In, but low radiolabelling yields were obtained for [^18^F]AlF owed to the N_3_O_3_ donor set; despite this, tumours could be visualised with [^18^F]AlF-NODAGA-E[c(RGDfK)]_2_ (3.44 ± 0.20%ID/g at 2 h p.i.) but the uptake was significantly lower than its ^68^Ga (6.23 ± 0.76%ID/g) and ^111^In (4.99 ± 0.64%ID/g) counterparts (Dijkgraaf et al. 2013). Integrin ⍺_v_β_6_ is expressed on the surface of epithelial cells in a variety of cancer types and can be targeted by the peptide A20FMDV2 (Bandyopadhyay and Raghavan 2009). Hausner et al. (2014) modified peptide with a NOTA-PEG_28_ moiety and radiolabelled with [^18^F]AlF complex ([^18^F]AlF-NOTA-PEG_28_-A20FMDV2) in a RCY (d.c.) of 19.3 ± 5.4% and a molar activity of 158 ± 36 GBq/µmol; the radioconjugate exhibited high cellular internalisation in ⍺_v_β_6_ positive cells and specific in vivo uptake.

### Imaging the gastrin-releasing peptide receptor (GRPR)

**Fig. 10:**
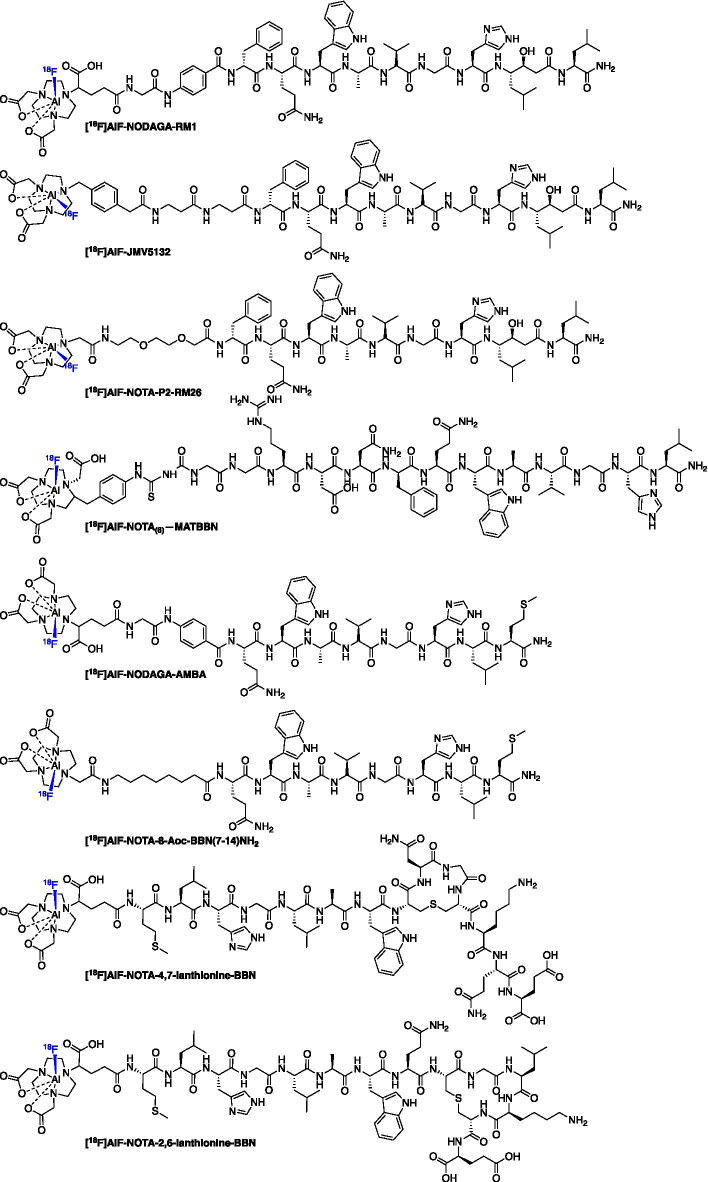
Structures of [^18^F]AlF BBN analogs targeting GRPR

The gastrin-releasing peptide receptor (GRPR) is a G protein-coupled receptor aberrantly expressed in some cancers, including prostate, colon and lung (Jensen et al. 2008), and numerous [^18^F]AlF-based radioligands have been developed based around bombesin (BBN) analogues (Fig. 10), either full-length or truncated sequences, which bind to GRPR with a high selectivity and affinity. The instability of BBN radioconjugates have limited the clinical translation of these probes (Vincentis et al. 2002, 2004; Scopinaro et al. 2003; Wiele et al. 2001, 2008; Ananias et al. 2013). Table 6 lists all probes discussed in this section along with their serum stability, tumour-to-blood (T:B) and tumour-to-muscle (T:M) ratios where available.

**Table 6:** A summary of GRPR targeted radiopharmaceuticals and their serum stabilities, tumour-to-blood (T:B) and tumour-to-muscle (T:M) ratios at a given time point post-injection

[^18^F]AlF radioconjugate	Serum stability at 1 h (%)^a^	T:B ratio	T:M ratio	Time point p.i. (h)^b^	References
[^18^F]AlF-NODAGA-RM1	> 90	10.29	14.58	2	Liu et al. (2013)
[^18^F]AlF-NODAGA-AMBA	72.5	–	8^c^	2	Liu et al. (2013)
[^18^F]AlF-NOTA-8-Aoc-BBN(7–14)NH_2_	–	49.51	–	1	Dijkgraaf et al. (2012)
[^18^F]AlF-NOTA-4,7-lanthionine-BBN	> 80 (human serum)	0.71	9.11	2	Carlucci et al. (2015)
[^18^F]AlF-NOTA-2,6-lanthionine-BBN	> 80 (human serum)	1.24	6.7	2	Carlucci et al. (2015)
[^18^F]AlF-NOTA_(6)_-MATBBN	> 95 (human serum)	9.44	30.70	1	Pan et al. (2014)
[^18^F]AlF-JMV5132	–	106	305^c^	1	Chatalic et al. (2014)
[^18^F]AlF-NOTA-P2-RM26	> 98	87	159	3	Varasteh et al. (2013)

^a^Intact parent radioconjugate in mouse serum after 1 h incubation, unless stated otherwise

^b^The timepoint post-injection where T:B and T:M data is reported. T:B = tumour-to-blood ratio and T:M = tumour-to-muscle ratio calculated from ex vivo biodistribution data presented in the original manuscript and represented as a mean value

^c^Estimated from graphical data in the original manuscript

Dijkgraaf et al. (2012) first described an [^18^F]AlF labelled NOTA-conjugated BBN analogue ([^18^F]AlF-NOTA-8-Aoc-BBN(7–14)NH_2_) which was produced in a RCY > 50% and molar activity > 10 GBq/µmol within 45 min. Uptake in PC-3 tumours at 1 h p.i. was 2.15 ± 0.55%ID/g and the signal was confirmed to be specific by co-injection of a blocking dose of NOTA-8-Aoc-BBN(7–14)NH_2_ (Dijkgraaf et al. 2012). Liu et al. (2013) synthesised [^18^F]AlF-NODAGA-RM1 and [^18^F]AlF-NODAGA-AMBA analogues. The [^18^F]AlF-NODAGA-RM1 and [^18^F]AlF-NODAGA-AMBA peptides were labelled in 40 min with a radiochemical yield of 5.6 ± 1.1% and 4.9 ± 1.3%, respectively; the molar activity was > 1.85 GBq/µmol (Liu et al. 2013). The [^18^F]AlF-NODAGA-RM1 radioligand was most stable in serum whereas [^18^F]AlF-NODAGA-AMBA degraded. Both [^18^F]AlF-NODAGA-RM1 and [^18^F]AlF-NODAGA-AMBA exhibited excellent in vivo tumour uptake (3.70 ± 0.70 and 4.60 ± 1.50%ID/g at 0.5 h) with [^18^F]AlF-NODAGA-RM1 showing significantly higher uptake than [^18^F]AlF-NODAGA-AMBA at 1 and 2 h p.i. time points (Liu et al. 2013). The authors conclude that [^18^F]AlF-NODAGA-RM1 is the most promising radioligand. Full-length BBN peptides are poorly stable in vivo so efforts were made by Carlucci et al. (2015) to stabilise the peptide using peptidase resistant lanthionine thioether crosslinked amino acids. Two radioconjugates were synthesised, [^18^F]AlF-NOTA-4,7-lanthionine-BBN and [^18^F]AlF-NOTA-2,6-lanthionine-BBN, which were produced in a RCY of 50–60% (d.c) with a molar activity > 63 GBq/µmol and > 88 GBq/µmol, respectively (Carlucci et al. 2015). Tumour uptake of both [^18^F]AlF-NOTA-4,7-lanthionine-BBN and [^18^F]AlF-NOTA-2,6-lanthionine-BBN in PC-3 xenografts at 120 min p.i. was 0.82 ± 0.23 and 1.40 ± 0.81%ID/g, respectively; moreover, both radioconjugates exhibited high in vivo stability with an average of 87–88% intact tracer in the tumour, confirming that lanthionine crosslinkers were advantageous (Carlucci et al. 2015). Introducing a hydrophilic linker into BBN analogues, as demonstrated by Pan et al. (2014) in their report of [^18^F]AlF-NOTA_(6)_-MATBBN can improve the PK profile of the radioconjugate and lead to high tumour uptake (4.59 ± 0.40%ID/g at 60 min p.i.) and excellent T:M contrast (6.77 ± 1.10) by image analysis. [^18^F]AlF-NOTA_(6)_-MATBBN was produced in a RCY of 62.5 ± 2.1% with a molar activity of at least 30 GBq/µmol (Pan et al. 2014).

The antagonist [^18^F]AlF-JMV5132 was produced in a RCY of 88% (n.d.c) with a molar activity of 40 ± 4 GBq/µmol which showed high in vivo uptake in PC3 tumours at 60 min p.i. (4.96 ± 1.20%ID/g) (Chatalic et al. 2014). Varasteh et al. (2013) developed [^18^F]AlF-NOTA-P2-RM26 a antagonist BBN analogue produced in a RCY of 60–65% (d.c.) and with a molar activity of 55 GBq/µmol (Varasteh et al. 2013); tumour uptake was high at 3 h p.i. (5.5 ± 0.7%ID/g) along with excellent T:B (87 ± 42) and T:M (159 ± 47) contrast (Varasteh et al. 2013).

### Imaging chemokine receptor CXCR4

**Fig. 11:**
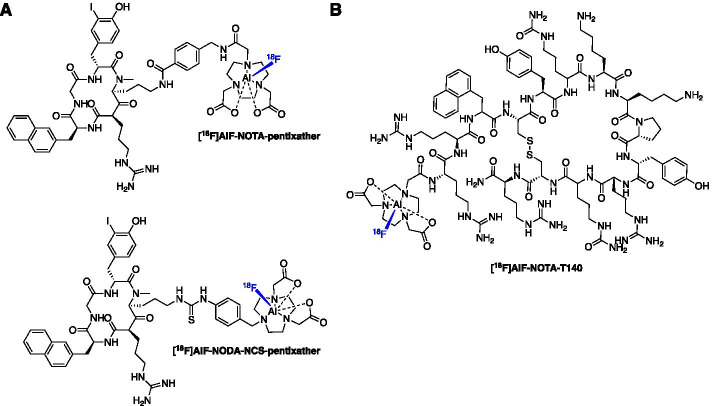
Structures of **A** [^18^F]AlF-pentixather derivatives and **B** [^18^F]AlF-NOTA-T140

Many cancers overexpress the chemokine receptor CXCR4 and therefore targeted diagnostic/prognostic imaging tools and therapeutics have been developed. The gallium-68 labelled peptide [^68^Ga]DOTA-pentixafor has been evaluated clinically in a variety of cancer types (Lapa et al. 2016; Herrmann et al. 2015; Philipp-Abbrederis et al. 2015; Wester et al. 2015). The [^18^F]AlF method was used to synthesise the first fluorine-18 labelled pentixafor-based agents [^18^F]AlF-NOTA-pentixather and [^18^F]AlF-NODA-NCS-pentixather. These radioconjugates were produced in a RCY of 45.5 ± 13.3% and a molar activity of up to 24.8 GBq/µmol (Fig. 11A) (Poschenrieder et al. 2016). Interestingly, the NODA chelator diminished the affinity of the peptide whereas the NOTA chelator was well tolerated and improved (1.4-fold improvement) affinity to CXCR4 as well as increased internalisation (threefold) compared to [^nat^Ga]DOTA-pentixafor (Poschenrieder et al. 2016). However, the lipophilicity showed negative impact on the biodistribution compared to [^68^Ga]DOTA-pentixafor. Yan et al. (2016) produced [^18^F]AlF-NOTA-T140 in a 58.0 ± 5.3% RCY and molar activity of 18.9 ± 1.1 GBq/µmol within 30 min (Fig. 11B). Tumour uptake positively correlated with CXCR4 expression with 26-fold greater uptake in CXCR4 positive vs. negative tumours (9.20 ± 2.08 vs. 0.33 ± 0.03%ID/g).

## Current and future perspectives on the [^18^F]AlF method

**Fig. 12:**
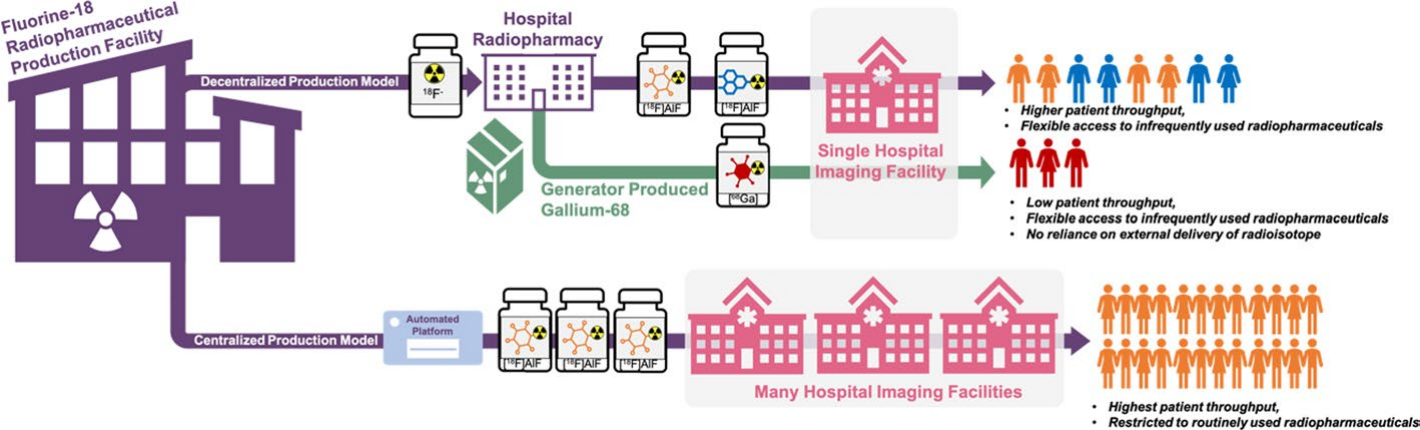
Whether [^18^F]AlF radiopharmaceuticals are produced in a decentralised radiopharmacy using kit-based procedures alongside ^68^Ga-radiopharmaceuticals, or a centralised fluorine-18 radiopharmaceutical production facility will depend on radiopharmaceutical demand and flexibility

The [^18^F]AlF method conveniently combines the favourable decay characteristics of fluorine-18 with the convenience of metal-based radiochemistry, as highlighted throughout this review. The avid adoption, implementation and continual development of the [^18^F]AlF method is a testament to the positive contribution it has made to radiopharmaceutical development. The [^18^F]AlF method solves two problems associated with gallium-68 chelation and “classical” carbon-^18^F-fluorine radiochemistry (direct fluorination and prosthetic group strategies):

1. ***A “complex” relationship with gallium-68*** Generator-produced gallium-68 (t_1/2_ = 68 min, β_em_^+^ = 89%) is used primarily to produce doses of PET radiopharmaceuticals from the local hospital radiopharmacy via a decentralised production model (Fig. 12). This is in contrast to the centralised production model exemplified by ^18^F-radiopharmaceuticals like [^18^F]FDG, which are produced in a large scale to serve several hospitals and imaging facilities from a single radiopharmaceutical production facility (Fig. 12). Gallium-68 metal-based radiochemistry is convenient and depending upon the age of the ^68^Ge/^68^Ga generator, can produce up to 3 patient doses per elution; increasing for high-capacity generators and cyclotron produced ^68^Ga. Although some future developments may increase this capacity the shorter half-life of gallium-68 remains an issue for a centralised supply model. As we implement personalised medicine into routine clinical practice and use PET biomarkers to underpin patient stratification and monitor treatment response, we need expedient solutions to scale radiopharmaceutical production to meet future clinical demand. The dose capacity of ^68^Ge/^68^Ga generators diminishes over time and is limited, which may be a concern if more ^68^Ga-radiopharmaceuticals gain approval for routine clinical use. Additionally, while it has not been fully evaluated and is somewhat context dependent, the variable and diminishing molar activity (A_m_) of ^68^Ga over the lifespan of a generator may confound PET imaging of some biological targets. Finally, the availability of generators has been a challenge in recent years with long lead-times on delivery and rising cost, both of which tighten the already slim financial margin for operating a sustainable radiopharmacy (Mueller et al. 2019). To futureproof the throughput for producing a repertoire of ^68^Ga-radiopharmaceuticals for the clinic, there is an option to commit resource to cyclotron produced gallium-68 where viable and effective methods have been described, producing > 3.5 GBq of ^68^Ga within 60 min from a liquid target with a 14.3 MeV beam energy (Rodnick et al. 2020); or, alternatively, we could develop ^18^F derivatives of commonly used ^68^Ga radiopharmaceuticals to harness the abundance of nucleophilic [^18^F]fluoride from existing radiopharmaceutical networks already producing [^18^F]FDG; this allows [^18^F]AlF derivatives to modulate tracer demand and would allow decentralised ^68^Ga production to focus on smaller batch runs for less frequent speciality tracer scans for lower numbers of patients. The [^18^F]AlF method promotes the latter without significant reimagination of existing radiopharmaceuticals, as would be necessary for most ^18^F-fluorination chemistries. It is promising that early data suggests ^68^Ga and [^18^F]AlF radiopharmaceuticals exhibit similar pharmacokinetic profiles (PK) although in some cases, differences have been observed, particularly in biodistribution (Hou et al. 2020).
2. ***Fluorine-18 radiochemistry is challenging!*** If we are to seek a centralised production model to produce ^18^F-peptides previously labelled with ^68^Ga, then a lot of development work is required. As we alluded to, developing fluorine-18 radiochemistry to radiolabel complex and sensitive molecules like proteins and peptides is challenging. The radiochemistry can be laborious and as the overall pharmacokinetic (PK) and metabolic profile is influenced by the radiolabelling and linker strategy, an iterative approach to developing suitable radioconjugates may be required which adds complexity. Several late-stage fluorination methods and ^18^F-prosthetic group strategies suitable for radiolabelling peptides have been described and some evaluated in the clinic, but none as close in characteristics to the existing ^68^Ga radiochemistry as the [^18^F]AlF method (Allott et al. 2020; Narayanam et al. 2020; Kee et al. 2020; Yuan et al. 2018; Krishnan et al. 2017; Cole et al. 2014). Therefore, if the research experience lies with gallium-68 radiochemistry, the parallels offered by the [^18^F]AlF method will be a facile adjustment.

Conventional fluorine-18 radiopharmaceuticals require automated radiosynthesis procedures for their GMP production, which adds additional complexity to the translational pathway (Allott and Aboagye 2020). However, automated procedures are convenient for [^18^F]AlF-based radiopharmaceuticals and will be necessary where a centralized production model produces multi-patient doses for transportation to hospitals from a remote facility (Fig. 12). Automation is not a necessity for low level production runs to access a couple of patient doses and therefore [^18^F]AlF is also amendable to decentralised, manual kit-based production akin to ^68^Ga-radiopharmaceuticals (Fig. 12). We often consider [^18^F]AlF as a replacement for ^68^Ga; the question *“Will* [^18^F]*AlF replace *^*68*^*Ga for metal chelate labelling?”* was presented in the title of an excellent review by Fersing et al. (2019) which provided a comprehensive overview of [^18^F]AlF radioconjugates, stopping short of speculating an answer to the question. The radiolabelling protocols for [^18^F]AlF and ^68^Ga are comparable and we have seen many examples where [^18^F]AlF-derivatives of ^68^Ga-peptides have been developed in order to address the limitations of ^68^Ga (i.e. short half-life and small dose batch sizes); but our opinion is that we *should not* consider [^18^F]AlF-radiopharmaceuticals as a replacement for ^68^Ga-radipharmaceuticals. ^68^Ga is, and always will be, a very important isotope that is unlikely to vanish from the clinic and actually, the flexibility that ^68^Ga will afford the clinic is yet to come into its own. The more personalised treatments we implement, the greater range of imaging radiopharmaceuticals we will require to stage and monitor disease, treatment efficacy and progression. This will inevitably lead to a large toolbox of radiopharmaceuticals, some of which will be used every day—as we see currently with [^18^F]FDG—where many patients benefit from a centralised production model which sets out to achieve multipatient and multihospital doses, allowing high patient throughput and in turn, lowers the cost-per-dose to healthcare services (Fig. 12); however, some radiopharmaceuticals may be used more infrequently, but their access is vital for patients with equivocal disease.

**Fig. 13:**
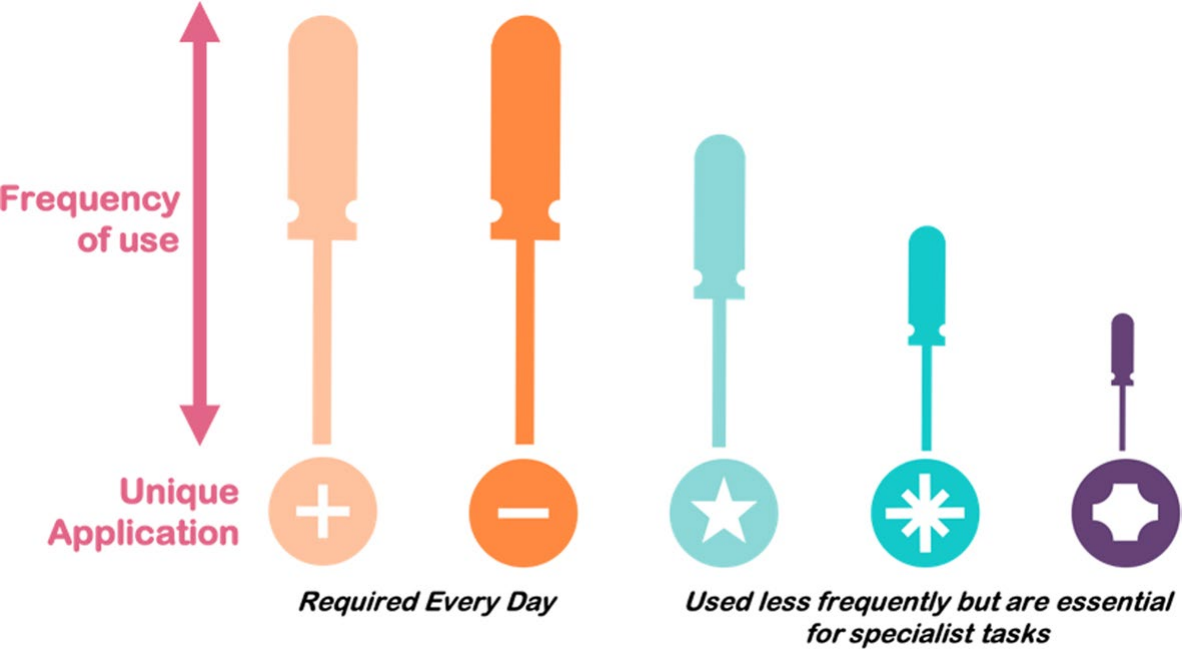
The screwdriver analogy. All tools are essential for the task they perform irrespective of how frequently that task is performed; the same is true for radiopharmaceuticals and therefore [^18^F]AlF and ^68^Ga radioconjugates both have an important role to play in modulating supply to meet the clinical demand for a range of imaging probes to offer patients the best possible care

Therefore [^18^F]AlF and ^68^Ga should be considered as complementary labelling feedstocks that can streamline radiopharmaceutical production to meet demand so that imaging facilities have the best possible reach for their patient population and disease types. We have analogized this concept to a set of screwdrivers, where all are essential to perform the operation for which they are designed, but some are required more frequently than others; demand does not correlate with importance in specific disease detection (Fig. 13). With this in mind, we envisage that multidose [^18^F]AlF radioconjugates will satisfy high demand imaging biomarkers and as a result, free up the radiopharmacy to produce small batches and a greater variety of ^68^Ga radioconjugates for lower demand imaging biomarkers. We consider that the complementary nature of [^18^F]AlF and ^68^Ga radiochemistry and their marginal differences in PET scan quality will not devalue ^68^Ga radiopharmaceuticals, but instead serves as a cohesive and powerful set of tools to improve the delivery of vital radiopharmaceuticals to the clinic.

### The future of [^18^F]AlF chelator design

We have an excellent variety of chelators to sequester [^18^F]AlF (Fig. 3); however, further expansion of ambient temperature chelators with improved stability is an area of research that could be further developed. Interestingly, a computational approach to calculate the thermodynamic stability of zirconium-89 chelates has been reported and we propose that this approach could be used to prospectively optimise the design of [^18^F]AlF chelators to focus synthesis efforts and experiment with underexplored functional groups (Holland 2020). The idea of developing more chelators can be supported or dismissed by the evaluation and clinical translation of [^18^F]AlF-PSMA-11, which presents an interesting conundrum! The HBED chelator is a poor choice for [^18^F]AlF chelation and results in a degree of in vivo demetallation; but, [^18^F]AlF-PSMA-11 is being pursued clinically for prostate cancer imaging, a disease renowned for osteoblastic lesions where the potential for the mischaracterisation of metastatic disease by off-target bone uptake may be high; based on this, one may argue that we can “make do” with the chelators we already have. On the other hand, perhaps [^18^F]AlF-PSMA-11 represents a scenario where we have placed the “horse before the cart” because the demand for [^18^F]AlF-based PSMA radiopharmaceuticals is such that we are willing to take the path of least resistance and accept the limitations of sub-optimal chelation because the necessary radiochemistry precursors are commercially available at this moment in time, to support clinical studies. We don’t think that this detracts from the implementation of optimal [^18^F]AlF chelators in our radioconjugates, but rather supports the idea of rapid commercialisation of clinically important precursors for the community to implement and evaluate.

With ever increasing interest in radionuclide therapy and theranostic isotope pairs, for example ^68^Ga and ^177^Lu which both form stable chelates with DOTA, should we now focus our attention to develop chelators to seamlessly support [^18^F]AlF and ^177^Lu isotope pairs from a single precursor molecule? There is a precedent for this approach in the literature (Lepage et al. 2020). Again, consideration needs to be given to future isotope requirements and, in this case, the impact of switching from an imaging isotope to a therapeutic isotope (where there may be future changes in demand).

### Improving accessibility to [^18^F]AlF-based radioconjugates

Automated methods are crucial if [^18^F]AlF-based radiopharmaceuticals are to be implemented within a centralized production model (Tshibangu et al. 2020; Allott et al. 2017). Despite extensive work in developing a raft of [^18^F]AlF-based radioligands for various targets, they are still not mainstream; an interesting communication by Hassan et al. (2019) discusses the challenge of implementing [^18^F]AlF-based radiopharmaceuticals in ‘normal’ research institutions or university hospitals even when NOTA-peptide conjugates are commercially available. They correctly highlight that the expertise required to implement the radiochemistry, and the manual nature [^18^F]AlF-radiochemistry reserves these radiopharmaceuticals for well-trained radiochemists and radiopharmacists in dedicated research institutions (Hassan et al. 2019). In time to come, we will see the commercialization of cassettes to produce [^18^F]AlF-radiopharmaceuticals on automated radiosynthesis platforms, simplifying the production of these products. This may not be the answer all production facilities are looking for, as automated radiochemistry required a large initial financial investment with full systems costing upwards of £100 k ($140 k), and continual maintenance and consumable overheads; however, automated radiochemistry has been significantly cost-reduced by the emergence of smaller platforms specifically designed for metal-based radiochemistry. Gallium-68 radioconjugates can now be produced from commercially available cassettes and we envisage that [^18^F]AlF-radioconjugates will follow suit. If production scale is not a concern, then commercial and validated kits may provide a convenient solution. New technologies like microfluidic “lab-on-a-chip” devices which have been exemplified for metal-based radiochemistry may provide automated radiochemistry and quality control on a single, inexpensive device for clinical use (He et al. 2017, 2016; Zhang et al. 2020). These devices may also serve to improve the issues of fluoride concentration and efficiency of [^18^F]AlF labelling (He et al. 2014; Wang and Dam 2020). While these are not fully established and are not yet commercially available, they may serve to bring safe, standardised and automated GMP production to facilities with less extensive infrastructure, staffed by nuclear medicine technologists.

## Conclusions

The [^18^F]AlF method has undoubtedly changed the landscape of fluorine-18 radiopharmaceutical development and adds another facet to metal-based radiolabelling that is relatively simple to implement and complements existing radiometal-based imaging agents. Over the last decade, the [^18^F]AlF method has risen to the status of an effective tool in radiopharmaceutical design and has all the characteristic hallmarks of longevity (i.e. implementation in new radiopharmaceutical development projects for a range of disease phenotypes, automated methods, extensive translation into clinical trials); while we cannot predict the future landscape of PET with any certainty, we hope to see [^18^F]AlF-based radiopharmaceuticals produced for the clinic on scale within the next 5–10 years.

## Abbreviations

RCY: Radiochemical yield
SA: Specific activity
A_m_: Molar activity
PK: Pharmacokinetic
d.c.: Decay corrected
n.d.c.: Non-decay corrected
