# Correction to: The aluminium-[18F]fuoride revolution: simple radiochemistry with a big impact for radiolabelled biomolecules

**DOI:** 10.1186/s41181-021-00148-7

**Published:** 2021-09-24

**Authors:** Stephen J. Archibald, Louis Allott

**Affiliations:** 1 Positron Emission Tomography Research Centre, Faculty of Health Sciences, University of Hull, Cottingham Road, Kingston upon Hull, HU6 7RX UK; 2 Department of Biomedical Sciences, Faculty of Health Sciences, University of Hull, Cottingham Road, Kingston upon Hull, HU6 7RX UK; 3 Hull University Teaching Hospitals NHS Trust, Castle Hill Hospital, Castle Road, Cottingham, HU16 5JQ UK

## Correction to: EJNMMI Radiopharm. Chem. (2021) 6:30 10.1186/s41181-021-00141-0

Following publication of the original article (Archibald and Allott 2021), the authors identified an error in Table 2 and Fig. 3. The correct table and figure are given below.

**Fig. 3:**
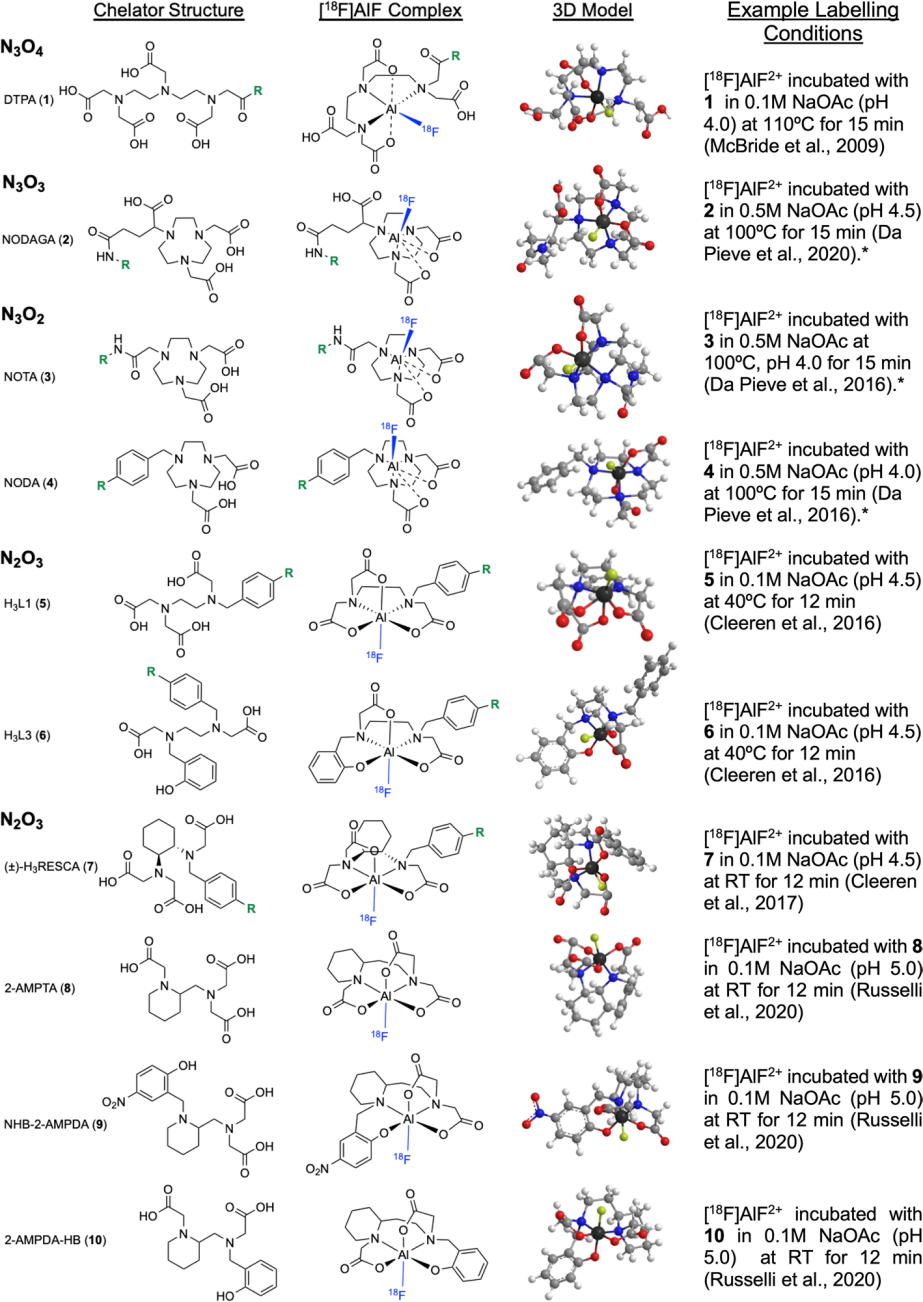
Structures of chelators evaluated for [ 18F]AlF and their proposed complexes. R = bioconjugation handle. 3D models were created in ChemBio3D (Cambridgesoft, UK) with MM2 energy minimization applied. Atom colours: carbon = light grey, hydrogen = white, oxygen = red, nitrogen = blue, fuorine = yellow, aluminium = dark grey. RT = room temperature. *Optional 1:1 (v/v) co-solvent included in the reaction mixture to improve RCY

The original article (Archibald and Allott 2021) has been corrected.

**Table 2:**
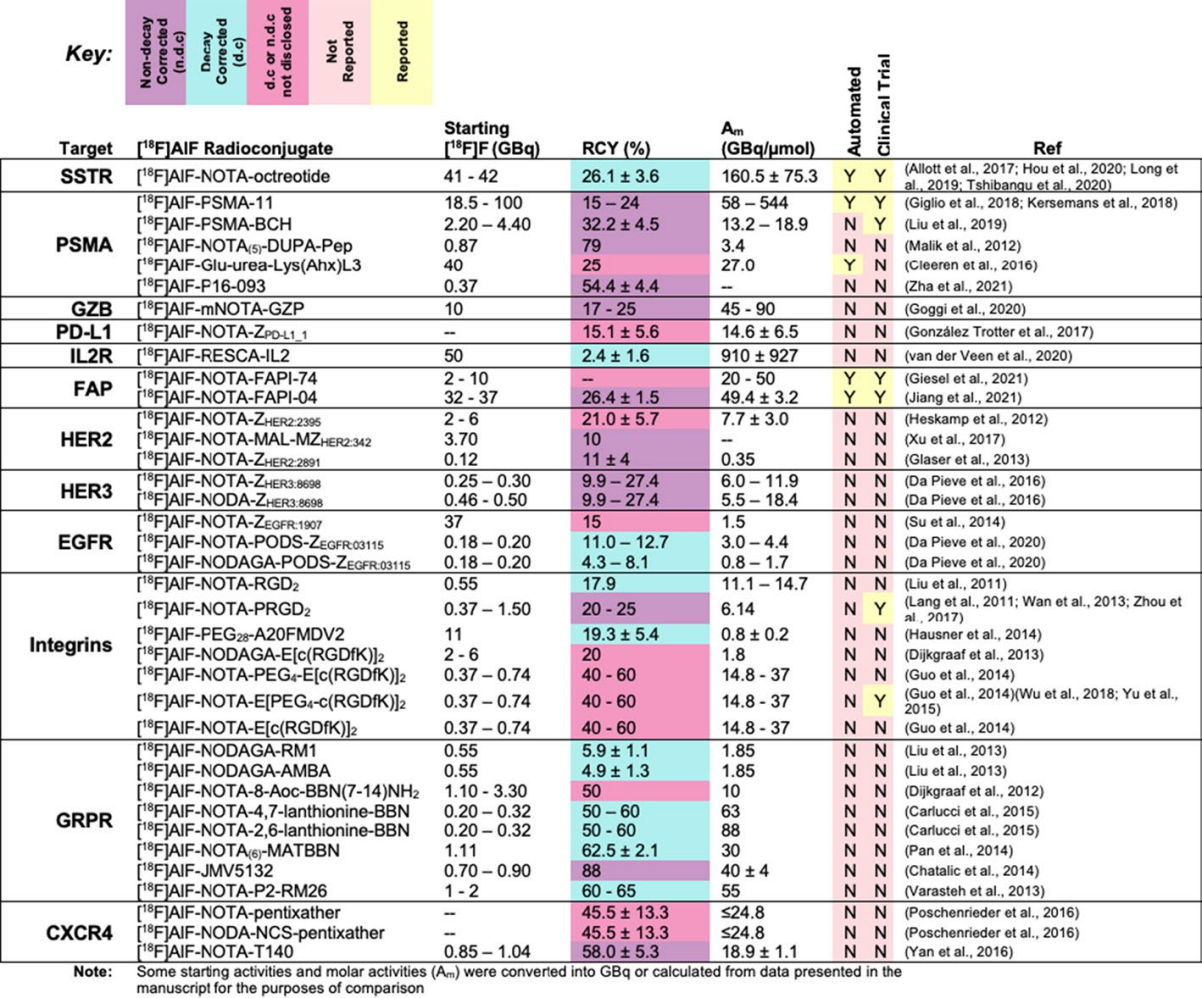
Prominent examples of [18F]AlF radioconjugates discussed in this review
